# Poly[aqua­bis­[μ_2_-2-(pyridin-4-ylsulfan­yl)acetato]­zinc]

**DOI:** 10.1107/S1600536811024512

**Published:** 2011-06-30

**Authors:** Zhi-Chao Wang, Bo Ding, Xiu-Guang Wang, Xiao-Jun Zhao

**Affiliations:** a College of Chemistry, Tianjin Key Laboratory of Structure and Performance for Functional Molecules, Tianjin Normal University, Tianjin 300387, People’s Republic of China

## Abstract

The crystal structure of the title complex, [Zn(C_7_H_6_NO_2_S)_2_(H_2_O)]_*n*_, consists of extended layers parallel to (001) with 2-(pyridin-4-ylsulfan­yl)acetate ligands bridging the Zn^II^ atoms. The Zn^II^ atom shows a distorted penta­gonal–bipyramidal coordination environment. The Zn^II^ and one O atom are situated on a crystallographic twofold rotation axis. In the crystal, intra­layer O—H⋯O hydrogen-bond inter­actions help to consolidate the coordination layer.

## Related literature

For metal complexes with polycarboxyl­ate aromatic ligands and their applications, see: Yang *et al.* (2007 ▶, 2010 ▶); Yu *et al.* (2010 ▶). For solid-state structures of metal complexes with pyridine-4-sulfanyl-acetate ligands, see Wang *et al.* (2011 ▶); Kondo *et al.* (2002 ▶).
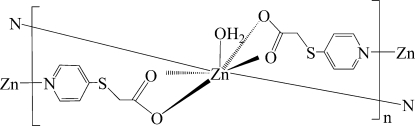


## Experimental

### 

#### Crystal data

[Zn(C_7_H_6_NO_2_S)_2_(H_2_O)]
*M* *_r_* = 419.76
Monoclinic, 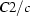

*a* = 16.057 (3) Å
*b* = 6.3709 (10) Å
*c* = 15.630 (3) Å
β = 95.393 (4)°
*V* = 1591.8 (5) Å^3^
*Z* = 4
Mo *K*α radiation
μ = 1.83 mm^−1^
*T* = 296 K
0.20 × 0.17 × 0.16 mm

#### Data collection

Bruker APEXII CCD area-detector diffractometer
Absorption correction: multi-scan (*SADABS*; Sheldrick, 1996 ▶) *T*_min_ = 0.711, *T*_max_ = 0.758
3842 measured reflections
1403 independent reflections
1308 reflections with *I* > 2σ(*I*)
*R*_int_ = 0.016

#### Refinement

*R*[*F* ^2^ > 2σ(*F* ^2^)] = 0.028
*wR*(*F* ^2^) = 0.072
*S* = 1.04
1403 reflections
110 parameters
H-atom parameters constrained
Δρ_max_ = 0.57 e Å^−3^
Δρ_min_ = −0.34 e Å^−3^

### 

Data collection: *APEX2* (Bruker, 2003 ▶); cell refinement: *SAINT* (Bruker, 2001 ▶); data reduction: *SAINT*; program(s) used to solve structure: *SHELXS97* (Sheldrick, 2008 ▶); program(s) used to refine structure: *SHELXL97* (Sheldrick, 2008 ▶); molecular graphics: *SHELXTL* (Sheldrick, 2008 ▶) and *DIAMOND* (Brandenburg & Berndt, 1999 ▶); software used to prepare material for publication: *SHELXL97*.

## Supplementary Material

Crystal structure: contains datablock(s) I, global. DOI: 10.1107/S1600536811024512/im2292sup1.cif

Structure factors: contains datablock(s) I. DOI: 10.1107/S1600536811024512/im2292Isup2.hkl

Additional supplementary materials:  crystallographic information; 3D view; checkCIF report

## Figures and Tables

**Table 1:** Hydrogen-bond geometry (Å, °)

*D*—H⋯*A*	*D*—H	H⋯*A*	*D*⋯*A*	*D*—H⋯*A*
O3—H3′⋯O1^i^	0.82	2.18	2.754 (3)	128

Symmetry code: (i) 
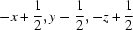
.

**Fig. 1.**
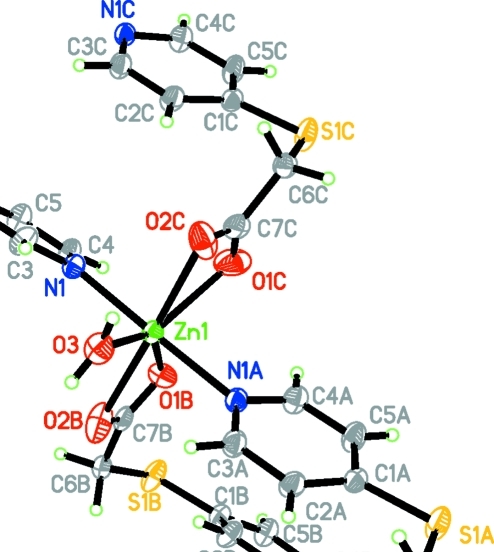
A portion of the two-dimensional structure of the title comlex. Displacement ellipsoids are drawn at the 30% probablity level. [Symmetry code: (A) 1 – x, y, 0.5 – z; (B) 1/2 + x, y, 0.5 – z; (C) 1/2 - x, 1/2 + y, 0.5 – z]

**Fig. 2.**
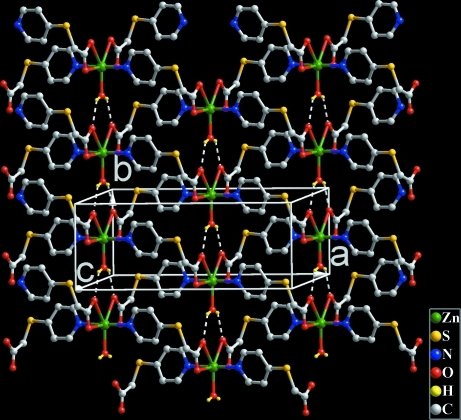
Part of the two-dimensional chain of (I), with hydrogen bonds shown as dashed lines.

## supplementary crystallographic information

### Comment

Recently, metal complexes constructed from aromatic polycarboxylate ligands and transition metal ions have received more and more interest due to their interesting architectures, amazing topologies and potentially technological applications in magnetism (Yang *et al.* 2010), luminesence (Yang *et al.* 2007) and gas storage (Yu *et al.* 2010). The anionic pyridine-4-sulfanyl-acetate ligand has three different potential binding sites upon coordination with metal ions and has exhibited various binding modes through the pyridyl N atom or carboxylate O donors. As a result, diverse complexes with discrete mononuclear (Wang *et al.* 2011), polymeric one-dimensional chains or two-dimensional layers (Kondo *et al.* 2002) have been obtained up to date. As a continuation of this research the crystal structure of a Zn^II^ complex with pyridine-4-sulfanyl-acetate ligands, (I), is reported herein.

A cut-out of the polymeric structure of the title compound showing one Zn^II^ atom in it's complete coordination environment is shown in Fig. 1. The Zn^II^ atom exhibits a distorted pentagonal bipyramidal coordination environment involving two pyridyl N atoms from two separate pyridine-4-sulfanyl-acetate ligands in *trans*-position, four O atoms from a pair of chelating carboxylate groups of pyridine-4-sulfanyl-acetate ligands and one O atom of a terminal water molecule. Each anionic pyridine-4-sulfanyl-acetate ligand acts as a ditopic connector to bridge adjacent Zn^II^ ions by a pyridyl N donor and a bidentate chelating carboxylate to generate a two-dimensional (4, 4) coordination layer with a Zn··· Zn distance of 6.3709 (10) Å. Additionally, O—H ···O hydrogen bonds between the coordinated water molecule and the deprotonated carboxylate (Table 1) help to consolidate the two-dimensional covalent layer (Fig. 2).

### Experimental

A methanolic solution of pyridine-4-sulfanyl-acetic acid (50.6 mg, 0.2 mmol) was carefully layered onto a buffer layer of ethyl acetate (2.0 ml) in a straight glass tube below which an aqueous solution containing Zn(NO_3_)_2_.6 H_2_O (44.6 mg, 0.15 mmol) was placed. The test tube was left in air at room temperature. Colorless block-shaped crystals were harvested within three weeks. Yield: 40% based on Zn^II^ salt. Anal. Calcd. for C_14_H_14_ZnN_2_O_5_S_2_: C, 40.06; H, 3.36; N, 6.67%. Found: C, 40.12; H, 3.26; N, 6.73%.

### Refinement

H atoms could be located from difference Fourier maps, but were subsequently placed in calculated positions and treated as riding, with C–H = 0.93 (aromatic), 0.97 (methylene) and O–H = 0.82 Å. All H atoms were allocated displacement parameters related to those of their parent atoms [*U_iso_*(H) = 1.2 *U_eq_*(C, O)].

### Crystal data

**Table:**

[Zn(C_7_H_6_NO_2_S)_2_(H_2_O)]	*F*(000) = 856
*M**_r_* = 419.76	*D*_x_ = 1.752 Mg m^−^^3^
Monoclinic, *C*2/*c*	Mo *K*α radiation, λ = 0.71073 Å
Hall symbol: -C 2yc	Cell parameters from 3018 reflections
*a* = 16.057 (3) Å	θ = 2.6–27.8°
*b* = 6.3709 (10) Å	µ = 1.83 mm^−^^1^
*c* = 15.630 (3) Å	*T* = 296 K
β = 95.393 (4)°	Block, colourless
*V* = 1591.8 (5) Å^3^	0.20 × 0.17 × 0.16 mm
*Z* = 4	

### Data collection

**Table:**

Bruker APEXII CCD area-detector diffractometer	1403 independent reflections
Radiation source: fine-focus sealed tube	1308 reflections with *I* > 2σ(*I*)
graphite	*R*_int_ = 0.016
φ and ω scans	θ_max_ = 25.0°, θ_min_ = 2.6°
Absorption correction: multi-scan (*SADABS*; Sheldrick, 1996)	*h* = −18→8
*T*_min_ = 0.711, *T*_max_ = 0.758	*k* = −7→7
3842 measured reflections	*l* = −17→18

### Refinement

**Table:**

Refinement on *F*^2^	Primary atom site location: structure-invariant direct methods
Least-squares matrix: full	Secondary atom site location: difference Fourier map
*R*[*F*^2^ > 2σ(*F*^2^)] = 0.028	Hydrogen site location: inferred from neighbouring sites
*wR*(*F*^2^) = 0.072	H-atom parameters constrained
*S* = 1.04	*w* = 1/[σ^2^(*F*_o_^2^) + (0.0333*P*)^2^ + 2.8608*P*] where *P* = (*F*_o_^2^ + 2*F*_c_^2^)/3
1403 reflections	(Δ/σ)_max_ < 0.001
110 parameters	Δρ_max_ = 0.57 e Å^−^^3^
0 restraints	Δρ_min_ = −0.34 e Å^−^^3^

### Special details

**Table:**

Geometry. All e.s.d.'s (except the e.s.d. in the dihedral angle between two l.s. planes) are estimated using the full covariance matrix. The cell e.s.d.'s are taken into account individually in the estimation of e.s.d.'s in distances, angles and torsion angles; correlations between e.s.d.'s in cell parameters are only used when they are defined by crystal symmetry. An approximate (isotropic) treatment of cell e.s.d.'s is used for estimating e.s.d.'s involving l.s. planes.
Refinement. Refinement of *F*^2^ against ALL reflections. The weighted *R*-factor *wR* and goodness of fit *S* are based on *F*^2^, conventional *R*-factors *R* are based on *F*, with *F* set to zero for negative *F*^2^. The threshold expression of *F*^2^ > σ(*F*^2^) is used only for calculating *R*-factors(gt) *etc*. and is not relevant to the choice of reflections for refinement. *R*-factors based on *F*^2^ are statistically about twice as large as those based on *F*, and *R*- factors based on ALL data will be even larger.

### Fractional atomic coordinates and isotropic or equivalent isotropic displacement parameters (Å^2^)

**Table:**

	*x*	*y*	*z*	*U*_iso_*/*U*_eq_	
Zn1	0.5000	0.96825 (6)	0.2500	0.03315 (15)	
S1	0.12091 (5)	0.98792 (11)	0.03086 (5)	0.0547 (2)	
N1	0.37305 (12)	0.9762 (3)	0.19086 (13)	0.0350 (4)	
O1	0.03140 (13)	0.7681 (4)	0.16602 (14)	0.0766 (7)	
O2	0.05159 (15)	0.4525 (4)	0.12413 (14)	0.0710 (7)	
O3	0.5000	0.6356 (4)	0.2500	0.0511 (7)	
H3'	0.4845	0.5927	0.2954	0.077*	
C1	0.21478 (15)	0.9755 (4)	0.09745 (16)	0.0366 (5)	
C2	0.24687 (15)	0.7966 (4)	0.13868 (17)	0.0429 (6)	
H2	0.2162	0.6726	0.1360	0.051*	
C3	0.32493 (15)	0.8043 (4)	0.18384 (17)	0.0445 (6)	
H3	0.3454	0.6825	0.2111	0.053*	
C4	0.34017 (16)	1.1504 (4)	0.15408 (17)	0.0432 (6)	
H4	0.3713	1.2735	0.1597	0.052*	
C5	0.26277 (16)	1.1574 (4)	0.10832 (18)	0.0458 (6)	
H5	0.2425	1.2835	0.0846	0.055*	
C6	0.08449 (15)	0.7230 (4)	0.02936 (15)	0.0384 (5)	
H6A	0.0389	0.7099	−0.0156	0.046*	
H6B	0.1294	0.6325	0.0142	0.046*	
C7	0.05446 (15)	0.6435 (5)	0.11276 (16)	0.0481 (7)	

### Atomic displacement parameters (Å^2^)

**Table:**

	*U*^11^	*U*^22^	*U*^33^	*U*^12^	*U*^13^	*U*^23^
Zn1	0.0299 (2)	0.0337 (2)	0.0348 (2)	0.000	−0.00208 (15)	0.000
S1	0.0409 (4)	0.0454 (4)	0.0725 (5)	−0.0058 (3)	−0.0227 (3)	0.0140 (3)
N1	0.0325 (10)	0.0370 (11)	0.0346 (10)	−0.0009 (8)	−0.0018 (8)	0.0017 (8)
O1	0.0574 (13)	0.121 (2)	0.0539 (12)	−0.0035 (13)	0.0178 (10)	−0.0277 (13)
O2	0.0762 (16)	0.0738 (16)	0.0580 (13)	−0.0330 (13)	−0.0205 (11)	0.0193 (11)
O3	0.0532 (16)	0.0350 (13)	0.0644 (17)	0.000	0.0022 (13)	0.000
C1	0.0305 (12)	0.0402 (13)	0.0383 (12)	−0.0005 (10)	−0.0013 (10)	0.0013 (10)
C2	0.0331 (13)	0.0370 (14)	0.0574 (15)	−0.0068 (10)	−0.0020 (11)	0.0082 (12)
C3	0.0362 (13)	0.0403 (14)	0.0554 (15)	−0.0010 (11)	−0.0040 (11)	0.0138 (12)
C4	0.0401 (13)	0.0344 (13)	0.0529 (15)	−0.0055 (11)	−0.0067 (11)	0.0005 (11)
C5	0.0428 (14)	0.0321 (13)	0.0598 (16)	0.0004 (11)	−0.0101 (12)	0.0059 (12)
C6	0.0336 (12)	0.0466 (14)	0.0341 (12)	−0.0047 (11)	−0.0023 (9)	−0.0056 (10)
C7	0.0284 (12)	0.077 (2)	0.0368 (13)	−0.0153 (13)	−0.0071 (10)	−0.0071 (14)

### Geometric parameters (Å, °)

**Table:**

Zn1—O3	2.119 (3)	O2—Zn1^iv^	2.208 (3)
Zn1—N1	2.158 (2)	O3—H3'	0.8200
Zn1—N1^i^	2.158 (2)	C1—C2	1.384 (3)
Zn1—O2^ii^	2.208 (3)	C1—C5	1.393 (3)
Zn1—O2^iii^	2.208 (3)	C2—C3	1.380 (3)
Zn1—O1^ii^	2.398 (3)	C2—H2	0.9300
Zn1—O1^iii^	2.398 (3)	C3—H3	0.9300
S1—C1	1.751 (2)	C4—C5	1.375 (4)
S1—C6	1.786 (3)	C4—H4	0.9300
N1—C4	1.336 (3)	C5—H5	0.9300
N1—C3	1.338 (3)	C6—C7	1.519 (4)
O1—C7	1.232 (4)	C6—H6A	0.9700
O1—Zn1^iv^	2.398 (3)	C6—H6B	0.9700
O2—C7	1.231 (4)		
			
O3—Zn1—N1	91.34 (5)	C7—O2—Zn1^iv^	96.1 (2)
O3—Zn1—N1^i^	91.34 (5)	Zn1—O3—H3'	109.5
N1—Zn1—N1^i^	177.32 (10)	C2—C1—C5	116.8 (2)
O3—Zn1—O2^ii^	87.40 (6)	C2—C1—S1	125.16 (19)
N1—Zn1—O2^ii^	87.95 (8)	C5—C1—S1	118.04 (19)
N1^i^—Zn1—O2^ii^	92.17 (8)	C3—C2—C1	119.3 (2)
O3—Zn1—O2^iii^	87.40 (6)	C3—C2—H2	120.4
N1—Zn1—O2^iii^	92.17 (8)	C1—C2—H2	120.4
N1^i^—Zn1—O2^iii^	87.96 (8)	N1—C3—C2	124.1 (2)
O2^ii^—Zn1—O2^iii^	174.79 (13)	N1—C3—H3	118.0
O3—Zn1—O1^ii^	142.81 (5)	C2—C3—H3	118.0
N1—Zn1—O1^ii^	88.69 (8)	N1—C4—C5	123.5 (2)
N1^i^—Zn1—O1^ii^	89.18 (7)	N1—C4—H4	118.2
O2^ii^—Zn1—O1^ii^	55.43 (8)	C5—C4—H4	118.2
O2^iii^—Zn1—O1^ii^	129.77 (8)	C4—C5—C1	119.9 (2)
O3—Zn1—O1^iii^	142.81 (5)	C4—C5—H5	120.0
N1—Zn1—O1^iii^	89.18 (7)	C1—C5—H5	120.0
N1^i^—Zn1—O1^iii^	88.69 (8)	C7—C6—S1	115.73 (18)
O2^ii^—Zn1—O1^iii^	129.78 (8)	C7—C6—H6A	108.3
O2^iii^—Zn1—O1^iii^	55.43 (8)	S1—C6—H6A	108.3
O1^ii^—Zn1—O1^iii^	74.38 (11)	C7—C6—H6B	108.3
C1—S1—C6	103.16 (11)	S1—C6—H6B	108.3
C4—N1—C3	116.3 (2)	H6A—C6—H6B	107.4
C4—N1—Zn1	121.53 (16)	O2—C7—O1	121.4 (3)
C3—N1—Zn1	122.05 (16)	O2—C7—C6	118.2 (3)
C7—O1—Zn1^iv^	87.0 (2)	O1—C7—C6	120.3 (3)
			
O3—Zn1—N1—C4	−163.91 (19)	C4—N1—C3—C2	2.8 (4)
N1^i^—Zn1—N1—C4	16.09 (19)	Zn1—N1—C3—C2	−172.8 (2)
O2^ii^—Zn1—N1—C4	108.7 (2)	C1—C2—C3—N1	0.0 (4)
O2^iii^—Zn1—N1—C4	−76.5 (2)	C3—N1—C4—C5	−2.4 (4)
O1^ii^—Zn1—N1—C4	53.3 (2)	Zn1—N1—C4—C5	173.3 (2)
O1^iii^—Zn1—N1—C4	−21.1 (2)	N1—C4—C5—C1	−0.9 (4)
O3—Zn1—N1—C3	11.5 (2)	C2—C1—C5—C4	3.7 (4)
N1^i^—Zn1—N1—C3	−168.5 (2)	S1—C1—C5—C4	−174.5 (2)
O2^ii^—Zn1—N1—C3	−75.8 (2)	C1—S1—C6—C7	70.1 (2)
O2^iii^—Zn1—N1—C3	99.0 (2)	Zn1^iv^—O2—C7—O1	−0.1 (3)
O1^ii^—Zn1—N1—C3	−131.3 (2)	Zn1^iv^—O2—C7—C6	−177.97 (17)
O1^iii^—Zn1—N1—C3	154.3 (2)	Zn1^iv^—O1—C7—O2	0.1 (3)
C6—S1—C1—C2	−1.8 (3)	Zn1^iv^—O1—C7—C6	177.9 (2)
C6—S1—C1—C5	176.2 (2)	S1—C6—C7—O2	−159.5 (2)
C5—C1—C2—C3	−3.2 (4)	S1—C6—C7—O1	22.6 (3)
S1—C1—C2—C3	174.8 (2)		

Symmetry codes: (i) −*x*+1, *y*, −*z*+1/2; (ii) −*x*+1/2, *y*+1/2, −*z*+1/2; (iii) *x*+1/2, *y*+1/2, *z*; (iv) *x*−1/2, *y*−1/2, *z*.

### Hydrogen-bond geometry (Å, °)

**Table:**

*D*—H···*A*	*D*—H	H···*A*	*D*···*A*	*D*—H···*A*
O3—H3'···O1^v^	0.82	2.18	2.754 (3)	128

Symmetry codes: (v) −*x*+1/2, *y*−1/2, −*z*+1/2.
